# Efficient visible-light photocatalytic degradation system assisted by conventional Pd catalysis

**DOI:** 10.1038/srep09561

**Published:** 2015-03-31

**Authors:** Yanlong Yu, Tao He, Lingju Guo, Yajun Yang, Limei Guo, Yue Tang, Yaan Cao

**Affiliations:** 1 Key laboratory of Weak-Light Nonlinear Photonics, Ministry of Education, TEDA Applied Physics Institute and School of Physics, Nankai University, Tianjin 300457, China; 2 Laboratory of Nanosystem and Hierarchical Fabrication, National Center for Nanoscience and Technology, Beijing 100190, China

## Abstract

Different approaches like doping and sensitization have been used to develop photocatalysts that can lead to high reactivity under visible-light illumination, which would allow efficient utilization of solar irradiation and even interior lighting. We demonstrated a conceptually different approach by changing reaction route via introducing the idea of conventional Pd catalysis used in cross-coupling reactions into photocatalysis. The –O–Pd–Cl surface species modified on Ni-doped TiO_2_ can play a role the same as that in chemical catalysis, resulting in remarkably enhanced photocatalytic activity under visible-light irradiation. For instance, Pd/Ni-TiO_2_ has much higher activity than N-TiO_2_ (about 3 ~ 9 times for all of the 4-XP systems) upon irradiation with wavelength of 420 nm. The catalytically active Pd(0) is achieved by reduction of photogenerated electrons from Ni-TiO_2_. Given high efficient, stable Pd catalysts or other suitable chemical catalysts, this concept may enable realization of the practical applications of photocatalysis.

In heterogeneous photocatalysis, the organic pollutants can be degraded via direct oxidization by the oxidative species generated upon photoexcitation of a photocatalyst. Many photocatalysts have been designed and prepared to achieve this. TiO_2_ is the most widely studied one due to its nontoxicity and high photostability^1^,^2^. However, so far the photocatalytic efficiency for all of these catalysts is still low, which keeps them far away from practical applications. It is noted that the efficiency of such a photocatalytic system (PCS) can be improved by controllable modulation of light harvest and behavior of photogenerated charge carriers, which can be realized by different design concepts, such as modulation of crystallographic facets and morphology, doping, sensitization, surface treatment, and combination with other semiconductors and noble metals^3^,^4^,^5^,^6^,^7^,^8^,^9^,^10^,^11^,^12^,^13^,^14^,^15^,^16^,^17^,^18^. The majority of these studies are focused on the modification of the photocatalysts themselves. However, the photodegration efficiency is still not high enough hitherto, especially for those under visible-light irradiation that accounts for about 44% of total solar energy. On the other hand, the efficiency can also be improved if the photodegradation route of pollutants can be changed beforehand. A different concept for the design of a PCS can be proposed accordingly.

It is known that the reaction rate can be promoted remarkably by a catalyst via changing the reaction route in a chemical reaction, as less free energy is required to reach the transition state (i.e., intermediate). Coupling reaction is one of the most famous catalytic chemical reactions, for which palladium (Pd) catalysts are often used^19^,^20^,^21^,^22^. The tetrakis(triphenylphosphine) palladium(0) is the common catalyst for homogeneous catalytic reaction, and in heterogeneous Pd catalysis, Pd is often fixed to a solid support in the form of dopant, particle, and complex^20^,^21^. Besides the coupling reactions, Pd catalysts can also be used in hydrogenation reaction^23^, hydrogen evolution^24^, and NO reduction^25^. Although the Pd species have been employed to improve the photocatalytic degradation too, the mechanism is different from the above Pd catalysis, for which the Pd species can act as the center for electron collection^26^, change active sites at the (grain) interface of two solids^27^, or absorb light due to surface plasma resonance effect^28^.

In this work, the concept of conventional Pd catalysis used in the cross-coupling reactions is thus introduced into the PCS so as to efficiently photodegrade the organic pollutant. The target molecules are the toxic 4-halogeno-phenol (X-C_6_H_4_-OH, denoted as 4-XP, X = F, Cl, Br, and I, respectively), as it exhibits similarity in molecular structure to those used in cross-coupling reactions and is very difficult to be removed from the contaminated water. The Pd catalyst is the –O–Pd–Cl surface species modified on Ni-doped TiO_2_. The obtained PCS shows greatly enhanced photodegradation of 4-XP under visible-light irradiation, implying the feasibility of this novel design concept for efficient photocatalysis.

## Results

### Structure and composition of as-prepared photocatalysts

**Figure 1:**
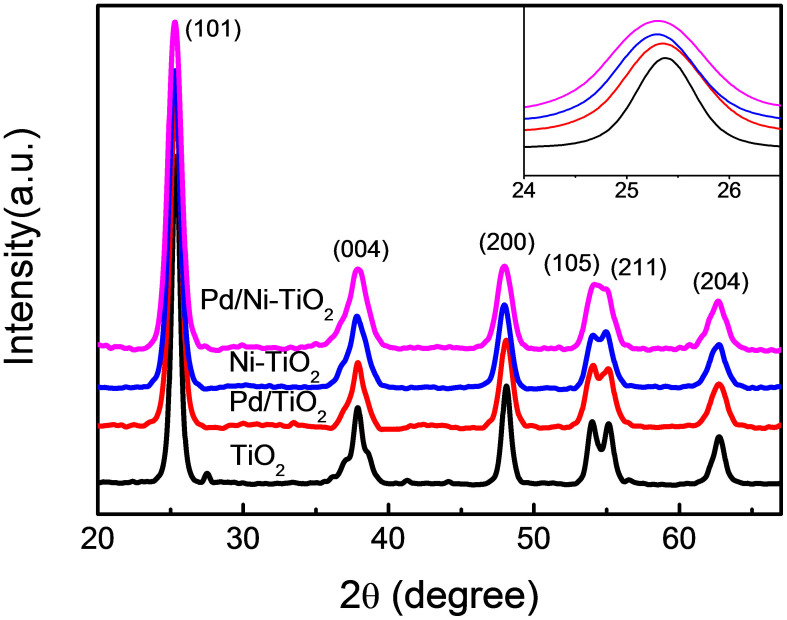
XRD patterns of different as-prepared photocatalysts. Inset is the enlarged XRD peaks of crystal plane (101).

The TiO_2_-based photocatalysts modified by Pd surface species are used in this work, which are prepared by a sol-gel method. Only anatase TiO_2_ can be observed in the XRD patterns for all of the photocatalysts (Fig. 1), indicating that no phase change among different samples. Moreover, no patterns ascribed to the oxides of Pd or Ni can be observed. The cell volume and lattice parameters can be derived from the diffraction peaks corresponding to crystal planes (101) and (200) in the XRD patterns using Scherrer's formula, for which of Ni-TiO_2_ are larger than those of pure TiO_2_ (Table 1). So Ni ions are doped in substitution mode as the ionic radius of Ni^2+^ (72 pm) is slightly larger than that of Ti^4+^ ions (68 pm). While the cell volume and lattice parameters remain almost unchanged upon Pd modification (i.e., almost no shift in XRD peak), implying that Pd ions are not present in substitution or interstitial mode since the ionic radius of Pd^2+^ (86 pm) is much larger than that of Ti^4+^ ions. This is further confirmed by the high-resolution TEM results (see Supplementary Fig. S1). The fringe spacing (d) of (101) crystallographic plane is determined to be 3.52 Å for both TiO_2_ and Pd/TiO_2_, indicating that the modified Pd exists as surface species; while it increases to 3.59 Å for Pd/Ni-TiO_2_, suggesting that Ni is doped into the crystal lattice as the ionic radius of Ni^2+^ is larger than that of Ti^4+^ ions. In addition, the BET specific surface area for TiO_2_, Ni-TiO_2_, Pd/TiO_2_, and Pd/Ni-TiO_2_ is determined to be 86.4, 77.0, 86.6, and 90.3 m^2^/g, respectively. That is to say, no big difference is found among different systems, which is consistent with the slight changes observed in the crystallite size (Table 1).

**Figure 2:**
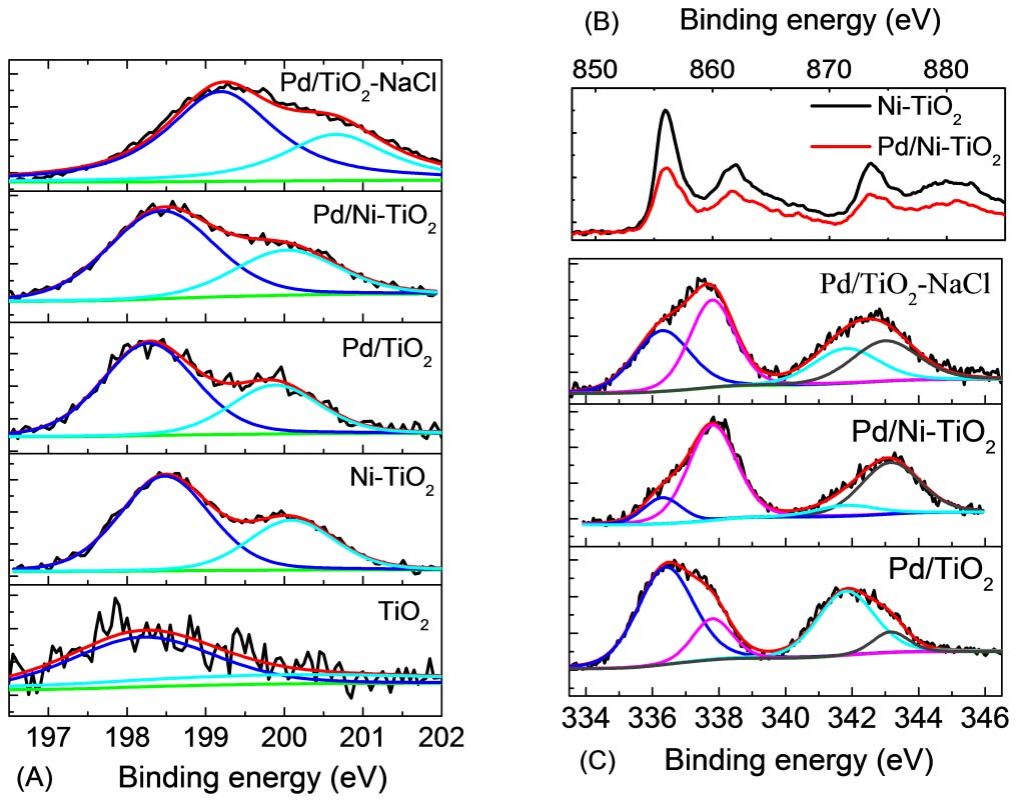
XPS spectra of different as-prepared photocatalysts. (A) Cl2p, (B) Ni2p and (C) Pd3d.

The chemical states of elements in different photocatalysts are determined by XPS technique. Since the radius of Cl^–^ ions (182 pm) is much larger than that of O_2_^–^ ions (140 pm), it is impossible for Cl^–^ ions to be doped into TiO_2_ lattice in substitution or interstitial mode. The peak at 198.1 eV for TiO_2_ sample is slightly lower than that of Cl2p_3/2_ in TiCl_4_ (Fig. 2A)^29^, which is ascribed to the Cl^–^ ions linked with unsaturated Ti sites on the surface of TiO_2_, i.e., –O–Ti–Cl structure^30^,^31^. Our previous work demonstrates that this –O–Ti–Cl cannot cause visible-light response and can hardly influence photocatalytic activity under visible-light irradiation^31^. For the Cl2p XPS spectra of Ni-TiO_2_, Pd/TiO_2_ and Pd/Ni-TiO_2_ samples, the binding energy (BE) of Cl2p_3/2_ peaks (198.3–198.5 eV) locates between that of TiCl_4_ (198.2 eV) and NiCl_2_ (199.2 eV) or PdCl_2_ (198.9 eV) (Fig. 2A). Hence, the Cl^–^ ions are linked with unsaturated Ti or other metal sites on the surface (i.e., –O–Me–Cl structure, Me = Ti, Ni, and Pd). The amount of such surface species increases greatly when TiO_2_ is modified by Ni and/or Pd, as the XPS signal increases significantly upon the modification.

For both Ni-TiO_2_ and Pd/Ni-TiO_2_ samples, two pairs of doublet Ni2p peaks as well as the corresponding satellite peaks (around 862 and 880 eV) can be observed in the spectra (Fig. 2B). The peak located at around 855.8 eV for Ni2p_3/2_ is attributed to the doped Ni ions in TiO_2_ lattice, since the peak position is almost the same as –Ti–O–Ni–O– structure in the bulk NiTiO_3_ (855.9 eV)^32^. Considering the above Cl2p XPS results, another one at around 856.7 eV is ascribed to the surface –O–Ni–Cl structure. Moreover, it seems that the total amount of Ni species in the catalyst decreases in the Pd/Ni-TiO_2_ compared with that in Ni-TiO_2_, as the XPS signal decreases obviously for Pd/Ni-TiO_2_.

Two pairs of doublet Pd3d peaks can be observed in the spectra for both Pd/TiO_2_ and Pd/Ni-TiO_2_ samples (Fig. 2C). The peak at 336.2 eV for Pd3d_5/2_ is ascribed to –O–Pd–O– structure at the surface (i.e., one Pd^2+^ ion is linked with two unsaturated oxygen ions). Considering the aforementioned Cl2p XPS results as well as the BE of Pd3d_5/2_ for PdO (336.3 eV) and PdCl_2_ (337.9 eV), another Pd3d_5/2_ peak at 337.7 eV is attributed to –O–Pd–Cl structure (i.e., one Pd^2+^ ion is linked with one Cl^–^ ion and one unsaturated oxygen ion) at the surface. Furthermore, the ratio of –O–Pd–Cl structure to –O–Pd–O– is much higher for Pd/Ni-TiO_2_ than Pd/TiO_2_, implying that the major surface species is –O–Pd–Cl for Pd/Ni-TiO_2_ while –O–Pd–O– for Pd/TiO_2_. Since the total amount of Ni species in Pd/Ni-TiO_2_ catalyst is less than that in Ni-TiO_2_, the utilization of Ni species during catalyst synthesis may promote the formation of surface –O–Pd–Cl species, possibly because the structure containing Ni^2+^ ions can change into –O–Pd–Cl in the presence of Pd^2+^ ions. So the synergistic interactions are present between Pd and Ni species to some degree during the catalyst synthesis.

In addition, NaCl, instead of NiCl_2_, has been used during the synthesis too, which can also provide Cl^–^ ions. The XPS signal of –O–Pd–Cl structure can be observed too. Moreover, the ratio of –O–Pd–Cl structure to –O–Pd–O– for the Pd/TiO_2_-NaCl catalyst is still larger than that for the Pd/TiO_2_, while it is smaller than that for Pd/Ni-TiO_2_ (Fig. 2C). This implies that the presence of Cl^–^ ions during the catalyst synthesis can be also in favor of the formation of surface –O–Pd–Cl species, especially via the conversion of –O–Pd–O– into –O–Pd–Cl, though the contribution may be less than that from the Ni species.

### Photocatalytic activity

**Figure 3:**
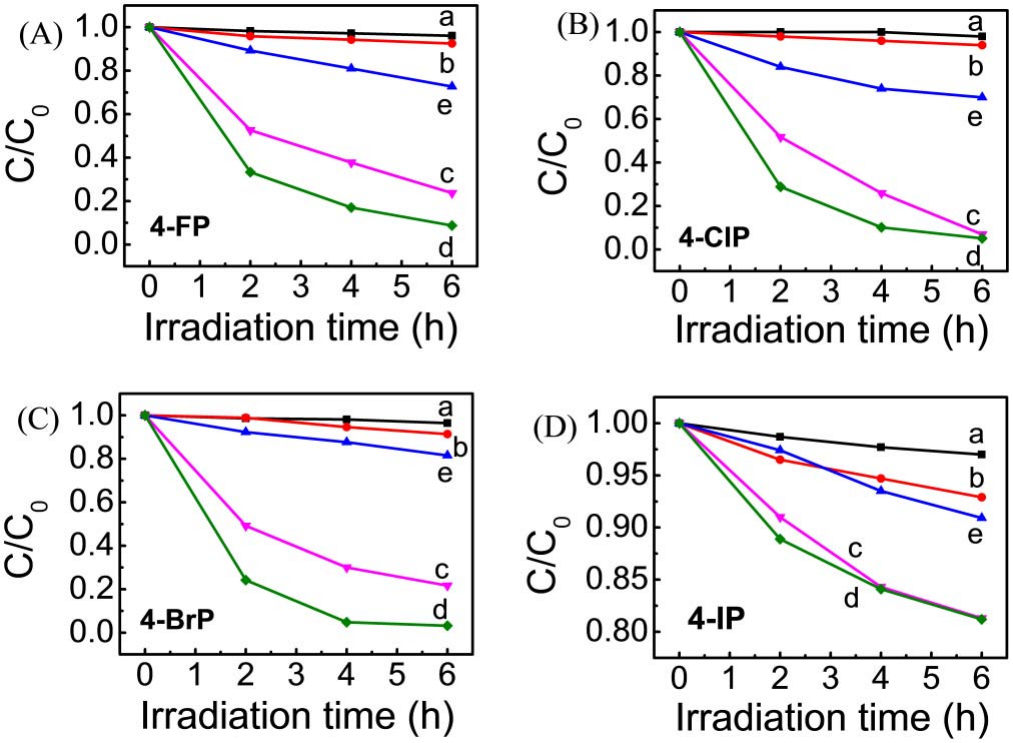
Photodegradation of different target molecules under visible-light irradiation (λ > 420 nm) in aqueous suspension with 10 mg of photocatalyst. (a) pure TiO_2_, (b) Ni-TiO_2_, (c) Pd/TiO_2_, (d) Pd/Ni-TiO_2_ and (e) N-TiO_2_.

Photodegradation of X-C_6_H_4_-OH (4-XP, X = F, Cl, Br, and I, respectively), a hazardous and toxic pollutant, has been used as target molecules to evaluate the photocatalytic activity of the resultant PCS under visible-light irradiation. The nitrogen doped TiO_2_ (N-TiO_2_) is also given here for comparison, as it shows relatively good visible-light photocatalytic activity. The photocatalytic activity upon visible-light irradiation (λ > 420 nm) falls in the series TiO_2_ < Ni-TiO_2_ < N-TiO_2_ < Pd/TiO_2_ < Pd/Ni-TiO_2_ for all of the systems (Fig. 3), especially in initial stage of the photodegradation. That is to say, TiO_2_ and Ni-TiO_2_ exhibit very low activity; while Pd/TiO_2_ shows a remarkably improved activity, and Pd/Ni-TiO_2_ has the highest activity. When compared Pd/Ni-TiO_2_ with Pd/TiO_2_ for the photodegradation in the first two hours, the enhancement amplitude is about 40.7%, 47.5%, 49.1%, and 23.3% for 4-FP, 4-ClP, 4-BrP, and 4-IP, respectively. Furthermore, Pd/Ni-TiO_2_ has much higher activity than N-TiO_2_ (about 3 ~ 9 times for all of the 4-XP systems), and is even much higher than Ni-TiO_2_ (about 15.3, 34.6, 67.9, and 2.2 times for 4-FP, 4-ClP, 4-BrP, and 4-IP, respectively). Thus, the presence of Pd species can greatly improve the visible-light photocatalytic activity of the reported PCS, while Ni species just improve it slightly. To further confirm this, in addition, 4-ClP has been photodegraded with less amount of photocatalyst (5 mg, instead of 10 mg in the above experiments) under illumination with slightly higher energy (λ > 400 nm). Exactly the same trend is observed (see Supplementary Fig. S2).

**Figure 4:**
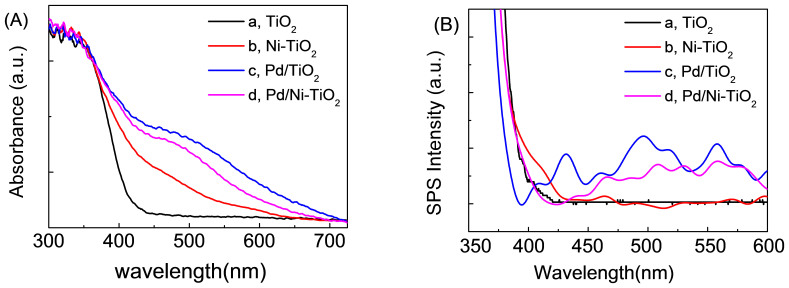
Optoelectronic properties of the as-prepared photocatalysts. (A) UV-vis absorption spectra and (B) surface photovoltaic spectra (SPS). (a) TiO_2_, (b) Ni-TiO_2_, (c) Pd/TiO_2_ and (d) Pd/Ni-TiO_2_.

Since no big difference in the BET specific surface area is found among TiO_2_, Ni-TiO_2_, Pd/TiO_2_, and Pd/Ni-TiO_2_, the effect of surface area on the photocatalytic activity can be ignored here. It is noted that the photocatalytic activity of a PCS is closely related to its optoelectronic properties. Pure TiO_2_ shows no visible-light absorption due to its large band gap of 3.1 eV, while the other three have relatively strong absorption in the visible-light region due to the formation of Ni doping energy levels and/or –O–Me–Cl surface species (Fig. 4A). It is noted that Pd/TiO_2_ has the strongest visible-light absorption, followed by Pd/Ni-TiO_2_. As discussed above, the major surface species is –O–Pd–Cl for Pd/Ni-TiO_2_ owing to the facilitated formation of –O–Pd–Cl by the presence of Ni species during catalyst synthesis, while it is –O–Pd–O– for Pd/TiO_2_. It is believed that –O–Pd–O– exhibits stronger visible-light absorption than –O–Pd–Cl and –O–Ni–Cl. This may explain why the Pd/TiO_2_ shows stronger visible-light absorption than Pd/Ni-TiO_2_.

Furthermore, the Pd/TiO_2_ exhibits higher visible-light surface photovoltaic spectroscopic (SPS) response throughout 400 ~ 600 nm than Pd/Ni-TiO_2_, although both of them are relatively low (Fig. 4B). Ni-TiO_2_ exhibits very little visible-light SPS response (only a small bump around 400 ~ 430 nm) and no visible-light SPS response at all for TiO_2_. Such a SPS response corresponds to the visible-light phtotocatalysis, i.e., only a PCS exhibits SPS response that can show photocatalysis, though maybe with different activity due to the influences from other factors. Besides the above photodegradation results, indeed, about 28.6% of 4-ClP are photodegraded for Pd/TiO_2_ and 33.3% for Pd/Ni-TiO_2_ under irradiation with a wavelength longer than 450 nm for 8 h, while no photodegradation is observed for Ni-TiO_2_. Then, the question arises. *Why the PCS with Pd/Ni-TiO_2_ shows lower visible-light absorption and SPS response than Pd/TiO_2_, but has higher photocatalytic activity*?

## Discussion

**Figure 5:**
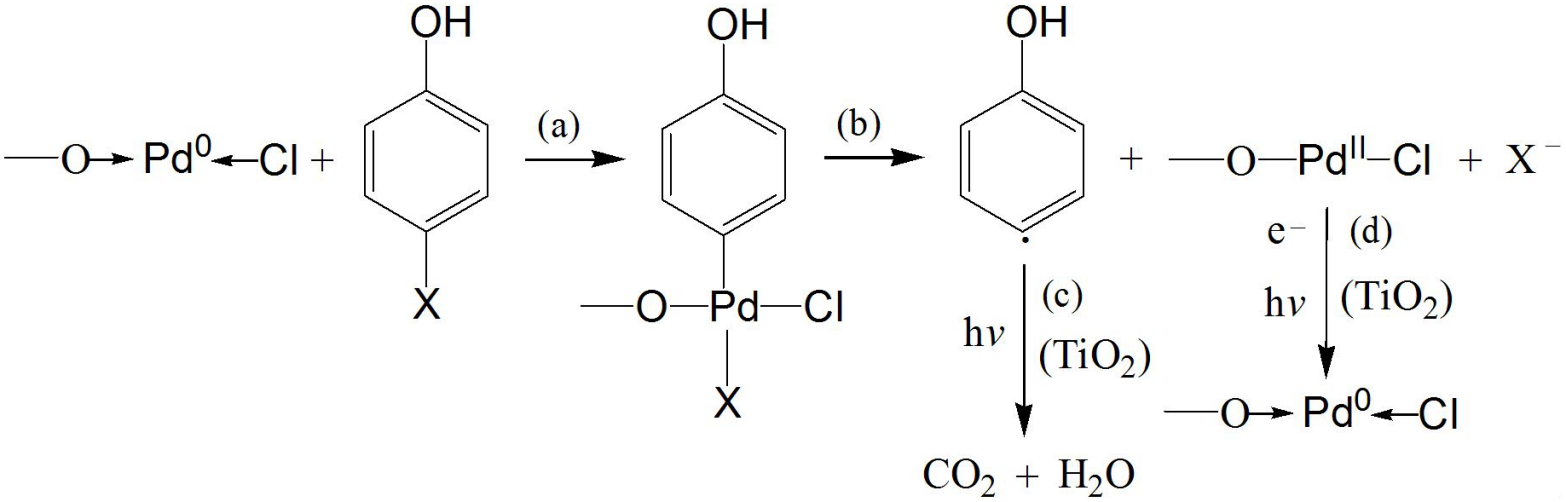
Photocatalytic degradation mechanism upon visible-light irradiation. X = F, Cl, Br, and I.

It is noted that the formation of some intermediates (such as hydroxyphenyl radicals) upon photoexcitation from the 4-XP molecules adsorbed on the photocatalyst surface is the first and critical step in the photodegradation, which will further undergo a sequence of reactions, eventually leading to a complete photodegradation of 4-XP into CO_2_ and H_2_O^33^,^34^. Thus, the photocatalytic efficiency can be improved significantly if the high free energy required for this critical process can be reduced via chemical catalysis. Considering the formation of a stable *trans*-*σ*-Pd(II) intermediate via reduction of a Pd(0) complex catalyst is the key step in the cross-coupling reactions^20^,^21^,^22^, it is suggested that the –O–Pd–Cl surface species reported here can play a similar role, i.e., acting as the catalyst too (actually –O→Pd(0) ←Cl). The catalytically active Pd(0) complex is formed *in-situ* in the cross-coupling reactions, typically through oxidation of a reductant like phosphine ligand if a Pd(II) catalyst is used initially. Since no such reducing agent is present in our PCS, it is suggested that here the active –O→Pd(0) ←Cl is formed via the reduction of –O–Pd(II)–Cl by photogenerated electrons upon irradiation, which can react with the target molecules to form organopalladium intermediates that can further produce the short-lived hydroxyphenyl radicals (HPR) via reaction with the photogenerated electrons and/or holes (Fig. 5). The degradation occurring thereafter is similar to those in conventional photocatalysis. It is believed that the conventional photodegradation mechanism plays a trivial role in this initial stage, even if it does play a role.

The formation of HPR has been confirmed previously^35^, which will undergo further rapid oxidation to the final mineralization products carbon dioxide and water. The regeneration of –O–Pd(II)–Cl from the active –O→Pd(0) ←Cl can be realized by the oxidization of oxygen or holes, though it is noted that the amount of –O–Pd(II)–Cl decreases to some degree according to the signal intensity of Cl2p XPS (see Supplementary Fig. S3). Except the 4-IP system, the relative reactivity in terms of photodegradation decreases in the order of Br > Cl > F, which is the same as that for the cross-coupling reactions. Moreover, in a moderate range, the photocatalytic activity increases with the increasing amount of Pd and Ni introduced during catalyst synthesis (see Supplementary Fig. S4), due to the increased amount of –O–Pd–Cl; while the activity decreases when the amount of Ni is too high, possibly because the surface Ni species can also act as recombination centers. Thus, the proposed concept is successfully realized, which may afford a feasible approach for design of an efficient PCS, even in the case of inferior visible-light absorption because the critical step for formation of the degradable intermediates is via Pd catalysis instead of via direct interactions with the photogenerated electrons or holes. This mechanism can also explain why the TiO_2_ modified by Ni and/or Pd has shorter photoluminescence lifetime than TiO_2_ (see Supplementary Fig. S5), while exhibits higher visible-light photodegradation activity on 4-XP as discussed above.

As for the 4-IP system, similar to that for the cross-coupling reactions, it is believed that the –O–Pd(II)–Cl may still exhibit very high catalytic activity for the formation of organopalladium intermediate and short-lived HPR, possibly higher than that for the 4-BrP too. Unlike the cross-coupling reactions, however, some of the resultant HPR can react with each other to form a dimer or other oligomer due to the absence of other reactants in the system for addition reactions with these intermediates. These oligomers are more difficult to be photodegraded than the corresponding monomer. Although no suitable facilities are available to probe the resultant intermediates or oligomers, this speculation can be proved indirectly by the UV-vis absorption spectra after irradiation (see Supplementary Fig. S6). The absorption corresponding to the benzene ring (~230 nm) decreases with increasing irradiation time. Meanwhile a new absorption bump appears at around 255 nm, corresponding to the absorption of oligomers. For the 4-IP system, the intensity of this new absorption reaches a maximum value in a relatively short time (2–3 h), while it keeps increasing with irradiation time for the 4-BrP system. This may suggest the rapid formation of oligomers for the 4-IP PCS due to high catalytic effect on the 4-IP system, resulting in the abnormal low photodegradation of 4-IP.

Here both the –O–Pd(II)–O– and –O–Ni(II)–Cl species cannot play such a role, or very little even if they could. For the former, it may be because it is difficult to form the organopalladium intermediate or HPR, possibly because both the oxygen ions linked to the Pd atom are also connected with another ion. Furthermore, PdO shows very low activity for the photocatalytic degradation of 4-XP (see Supplementary Fig. S7). On the other hand, no PdO is observed from the XRD patterns. For the –O–Ni(II)–Cl, it is possibly because it cannot be reduced efficiently by photogenerated electrons. Or, even if organonickel intermediate could be formed, calculation results indicate that the dissociation energy of Ni-X in the corresponding intermediate is larger than that of Pd-X (see Supplementary Table S1). So the X atom linked to the Pd atom can be more easily dropped out than that linked to Ni. So the –O–Ni(II)–Cl would not be an efficient catalyst in the 4-XP degradation, even it could act as a chemical catalyst. In addition, although no direct evidence shows the existence of the active –O→Pd(0) ←Cl intermediate under visible-light irradiation, the signal of Pd(0) can be observed in the XPS spectrum (~334.5 eV for Pd3d5/2) after UV-light photodegradation of target molecules when the same photocatalysts are used (see Supplementary Fig. S8). This may also imply the viability of the proposed mechanism.

**Figure 6:**
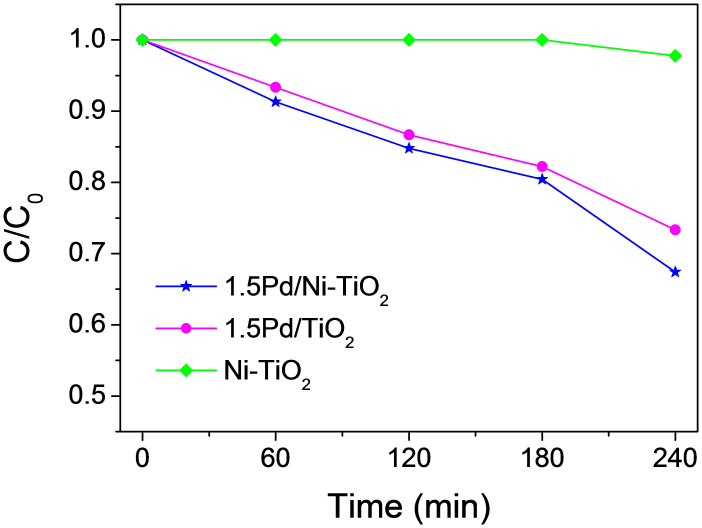
Photodegradation of 4-ClP under visible-light irradiation (λ > 420 nm) in aqueous suspension with 10 mg of different photocatalysts prepared without the presence of Cl species in the starting materials.

Moreover, as discussed above, the presence of Cl^–^ ions during the catalyst synthesis can be in favor of the formation of surface –O–Pd–Cl species, especially via the conversion of –O–Pd–O– into –O–Pd–Cl. Thus, the photocatalysts have also been prepared with the same protocol but without the presence of Cl species in the starting materials so as to further study the effect of Cl^-^ ions. It is found that all the obtained photocatalysts still exhibit visible-light response (see Supplementary Fig. S9). Similar to the catalysts prepared with the presence of Cl species, Pd/TiO_2_ has the strongest visible-light absorption, followed by Pd/Ni-TiO_2_, and Ni-TiO_2_ is the weakest. It can be seen from Figure 6 that the photodegradation activity of the obtained catalysts is much lower than the catalysts prepared with the presence of Cl species (Fig. 3b). For instance, the obtained Ni-TiO_2_ shows almost no activity on 4-ClP photodegradation. When the same amount of Pd is used in the precursor (1.5%), only about 35% of 4-ClP is decomposed after 4 hours of photodegradation for the catalyst prepared without Cl (1.5Pd/Ni-TiO_2_); while almost all of the 4-ClP is decomposed after 4 hours for the catalyst prepared with Cl (Fig. 3b). The same is true for the Pd/TiO_2_ catalyst. Therefore, the presence of Cl species in the precursor is very important. These results can further confirm the feasibility of the proposed mechanism.

## Conclusion

In summary, we have demonstrated a new design concept for efficient visible-light photocatalysis, i.e., a conventional Pd catalysis used in the coupling reactions is introduced into the PCS so as to change the photodegradation route of the pollutants. The results show that the Pd surface species modified on the Ni-doped TiO_2_ can act as the Pd catalyst. The catalytically active Pd(0) is achieved by reduction of photogenerated electrons from Ni-TiO_2_. The resultant PCS exhibits remarkably improved efficient photodegradation of 4-XP under visible-light irradiation. Stability, activity, selectivity and versatility of such Pd complex catalysts are open to the systematic study for developing suitable PCS so as to enable realization of the practical applications of photocatalysis. The Pd may be replaced by Ni, Fe or Cu in a specific system. We envision that the future investigations may even develop a PCS that can work efficiently in the case of inferior visible-light absorption, as the critical step for formation of the intermediates is via Pd catalysis instead of via direct interactions with the photogenerated electrons or holes. We believe that not only the field of photocatalytic degradation, but also other photocatalytic fields like photosynthesis may be benefited from this too since it is developed from coupling reactions.

## Methods

### Chemicals

All chemicals used were of analytical grade and the water was deionized water (>18.2 MΩ·cm). At room temperature, certain volume of NiCl_2_ (0.5 mol/L) and PdCl_2_ (0.1169 mol/L) solution were mixed with 40 mL of ethanol. Then 1 mL of HCl solution (12 mol/L) and 12 mL of Ti(OC_4_H_9_)_4_ was added dropwise into the mixture under vigorous stirring. The mixture was stirred until the formation of TiO_2_ gel, followed by being aged for 24 h. The obtained gels were dried at 373 K for 10 h and annealed at 723 K in a muffle for 2.5 h. The resultant samples were denoted as Pd/Ni-TiO_2_. Pure TiO_2_, Ni doped TiO_2_ (Ni-TiO_2_) and Pd modified TiO_2_ (Pd/TiO_2_) were prepared using the same procedure, while without the addition of corresponding precursor. To study the effect of Cl^-^ ions, PdSO_4_ and Ni(NO_3_)_2_ were also used as the precursors, for which the photocatalysts were prepared with the same protocol. Unless stated otherwise, the nominal molar ratio of Pd^2+^ to Ti^4+^ is fixed at 1.5% in the precursor and the one for Ni^2+^ to Ti^4+^ is 15%. For comparison, other molar ratio was also used for both Pd^2+^ to Ti^4+^ (such as 0.3%, 1.0%, and 1.3%) and Ni^2+^ to Ti^4+^ (such as 5%, 10%, and 20%).

### Characterization

The XRD patterns were acquired using a Rigaku D/max 2500 X-ray diffraction spectrometer (Cu Kα, λ = 1.54056 Å). The average crystallite size was calculated according to the Scherrer formula (*D* = k λ/*B* cosθ). XPS measurements were carried out by using a Thermo ESCALAB 250 spectrometer with an Al Kα monochromator source and all the binding energies were calibrated to the adventitious C1s peak at 284.8 eV. The specific surface area of the obtained catalysts were determined by Brunauer–Emmett–Teller (BET) analysis (Micromeritics, tristar II 3020). Diffuse reflectance UV-visible (UV-vis) absorption spectra were recorded on a UV-vis spectrometer (U-4100, Hitachi). The surface photovoltage spectroscopy (SPS) measurements were performed with a solid sandwich structure via a home-made system using a light source-monochromator lock-in detection technique.

### Calculation

All DFT calculations were performed using the DMol3 package^36^,^37^. The generalized gradient approximation (GGA) for the exchange-correlation potential prescribed by Perdew-Burke-Ernzerhof (PBE) and an all-electron double numerical basis set (DND) with polarization functions are used in spin-unrestricted density-functional-theory (DFT) based calculations^38^. For Pd atom, the relativistic effects were also considered. Geometry optimization was performed without symmetry constraint using a convergence criterion of 1.0 × 10^−5^ Hartree on the maximum energy gradient and 0.005 Å on the maximum displacement for each atom. Self-consistent field (SCF) electronic structure calculations were carried out with a convergence criterion of 1.0 × 10^−6^ Hartree on the total energy.

### Photocatalysis

The photocatalytic activity of all photocatalysts were determined by visible-light photodegradation of 4-XP solution (5 × 10^−5^ mol L^−1^, 40 mL) with a sunlamp (Philips HPA 400/30S, Belgium) and a cutoff filter for the removal of UV light. The reactor was perpendicular to the light beam and located 10 cm away from the light source. All the suspensions were magnetic stirred at (25 ± 2)°C in the dark for 30 min to reach adsorption equilibrium before photocatalysis, and oxygen gas was continuously bubbled through the suspension at a flux of 5 mL min^−1^. The change in concentration of 4-ClP was monitored by a UV-visible spectrometer (UV-1061PC, SHIMADZU) using 4-aminoantipyrine as the chromogenic reagent, while the degradation of 4-FP, 4-BrP and 4-IP was monitored directly by the spectrometer. The reproducibility of the photocatalytic degradation was evaluated by repeating experiments at least three times with different batches of photocatalysts prepared by the same procedure.

## Supplementary Material

Supplementary Information Supplementary information

## Figures and Tables

**Table 1:** Cell parameters, cell volume and crystallite size of different samples derived from XRD data given in Figure 1

*Sample*	*Cell parameters/Å*		*Cell volume/Å*^3^	*Crystallite size/nm*
	*a = b*	*c*		
TiO_2_	3.7760	9.4039	134.08	14.2
Pd/TiO_2_	3.7786	9.3955	134.15	10.6
Ni-TiO_2_	3.7903	9.4704	136.06	9.8
Pd/Ni-TiO_2_	3.7890	9.4914	136.26	9.5
