# Corrigendum: Efficient visible-light photocatalytic degradation system assisted by conventional Pd catalysis

**DOI:** 10.1038/srep46984

**Published:** 2018-05-14

**Authors:** Yanlong Yu, Tao He, Lingju Guo, Yajun Yang, Limei Guo, Yue Tang, Yaan Cao

Scientific Reports 5: Article number: 956110.1038/srep09561; published online: 03 31 2015; updated: 05 14 2018

This Article contains an error. The Authors stated that the cell volume and lattice parameters were derived using Scherrer’s formula. These parameters were actually calculated using Bragg’s law.

In the Results section,

“The cell volume and lattice parameters can be derived from the diffraction peaks corresponding to crystal planes (101) and (200) in the XRD patterns using Scherrer’s formula, for which of Ni-TiO_2_ are larger than those of pure TiO_2_ (Table 1).”

should read:

“The cell volume and lattice parameters can be derived from the diffraction peaks corresponding to crystal planes (101) and (200) in the XRD patterns using Bragg’s law, for which of Ni-TiO_2_ are larger than those of pure TiO_2_ (Table 1).”

This change does not affect the conclusions of the article. The authors apologize for the error.

