# Supplementary material for: Efficient visible-light photocatalytic degradation system assisted by conventional Pd catalysis
Source: Sci Rep. 2015 Mar 31;5:9561. doi: 10.1038/srep09561 (PMC5380333; doi:10.1038/srep09561)
Supplement: Supplementary Information [file srep09561-s1.doc]

*Electronic Supplementary Information*

Efficient Visible-Light Photocatalytic Degradation System Assisted by Conventional Pd Catalysis

Yanlong Yu,^a^ Tao He,*^b^ Lingju Guo,^b^ Yajun Yang,^a^ Limei Guo,^a^ Yue Tang^a^ and Yaan Cao*^a^

^a^ Key laboratory of Weak-Light Nonlinear Photonics, Ministry of Education, TEDA Applied Physics Institute and School of Physics, Nankai University, Tianjin 300457, China

^b^ Laboratory of Nanosystem and Hierarchical Fabrication, National Center for Nanoscience and Technology, Beijing 100190, China

**Figure S1.** HR-TEM images of different photocatalysts.

**Figure S2.** Photodegradation of different target molecules under visible-light irradiation (λ > 400 nm) in aqueous suspension with 5 mg of photocatalyst.

**Figure S3.** Cl 2p XPS spectra of Pd/TiO_2_ and Pd/Ni-TiO_2_ samples before and after photodegradation reaction.

**Figure S4.** Photodegradation rate of 4-ClP in aqueous suspension using different catalysts.

**Figure S5.** Time-resolved photoluminescence (TR-PL) decay curves for different catalysts, excited at 400 nm and monitored at 500 nm.

**Figure S6.** UV-Vis absorption spectra for photodegradation rate of 4-XP under visible light irradiation (λ > 420 nm) for different time with Pd/Ni-TiO_2_ catalyst. (A) 4-IP and (B) 4-BrP.

**Figure S7.** Photodegradation of different target molecules using PdO as the photocatalyst under visible-light irradiation (λ > 420 nm) in aqueous suspension.

**Figure S8.** Pd3d XPS spectrum after UV-light photodegradation of 4-BrP.

**Figure S9.** UV-vis absorption spectra of different photocatalysts prepared without the presence of Cl species in the starting materials.

**Table S1.** Calculated dissociation energy (Ed) of Pd-X in the HO-C_6_H_4_-PdCl(X) intermediate and Ni-X in the HO-C_6_H_4_-NiCl(X) intermediate.


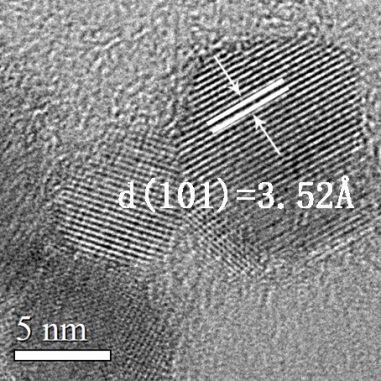

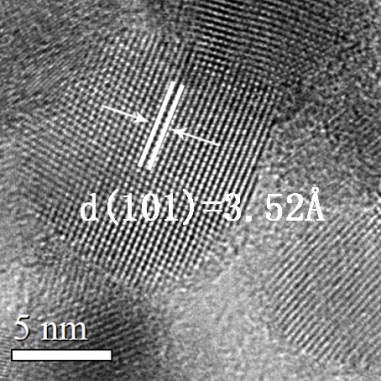

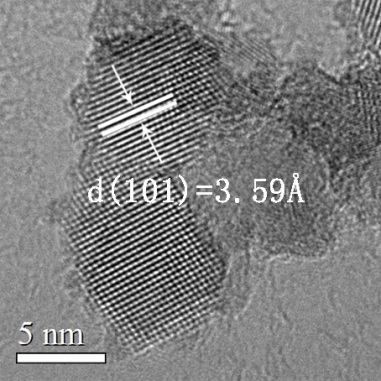


Pure TiO_2_ Pd-TiO_2_ Pd/Ni-TIO_2_

**Figure S1.** HR-TEM images of different photocatalysts.

**Figure S2.** Photodegradation of different target molecules under visible-light irradiation (λ > 400 nm) in aqueous suspension with 5 mg of photocatalyst. (a) pure TiO_2_, (b) Ni-TiO_2_, (c) Pd/TiO_2_, and (d) Pd/Ni-TiO_2_.

**Figure S3**. Cl 2p XPS spectra of (A) Pd/TiO_2_ and (B) Pd/Ni-TiO_2_ samples before and after photodegradation reaction.

**Figure S4**. Photodegradation rate of 4-ClP in aqueous suspension using different catalysts, (A) under visible light irradiation (λ > 420 nm) with 10 mg of photocatalyst for 2 h, and (B) under visible-light irradiation (λ > 400 nm) with 5 mg of photocatalyst for 4 h.

**Figure S5.** Time-resolved photoluminescence (TR-PL) decay curves for different catalysts, excited at 400 nm and monitored at 500 nm.

**Figure S6**. UV-Vis absorption spectra for photodegradation rate of 4-XP under visible light irradiation (λ > 420 nm) for different time with Pd/Ni-TiO_2_ catalyst. (A) 4-IP and (B) 4-BrP.

**Figure S7.** Photodegradation of different target molecules using PdO as the photocatalyst under visible-light irradiation (λ > 420 nm) in aqueous suspension.

**Figure S8.** Pd3d XPS spectrum after UV-light photodegradation of 4-BrP.

**Figure S9.** UV-vis absorption spectra of different photocatalysts prepared without the presence of Cl species in the starting materials.

**Table S1.** Calculated dissociation energy (Ed) of Pd-X in the HO-C_6_H_4_-PdCl(X) intermediate and Ni-X in the HO-C_6_H_4_-NiCl(X) intermediate. Here in the table the intermediate is simplified as Cl-Pd-X and Cl-Ni-X. X = F, Cl, Br, and I.

|  | Cl-Pd-F | Cl-Pd-Cl | Cl-Pd-Br | Cl-Pd-I | Cl-Ni-F | Cl-Ni-Cl | Cl-Ni-Br | Cl-Ni-I |
| --- | --- | --- | --- | --- | --- | --- | --- | --- |
| E_d_ (eV) | 3.486 | 2.776 | 2.438 | 1.937 | 4.518 | 3.501 | 2.941 | 2.433 |
